# Cataract surgery: ensuring equal access for boys and girls

**Published:** 2009-06

**Authors:** Annie Bronsard, Sylvia Shirima

**Affiliations:** PhD Candidate, University Laval; Policy Analyst, Health Canada. Email: annie_bronsard@hc-sc.gc.ca; Project Coordinator, Kilimanjaro Centre for Community Ophthalmology, PO Box 2254, Moshi, Tanzania. Email: sshirima@kcco.net

**Figure:**
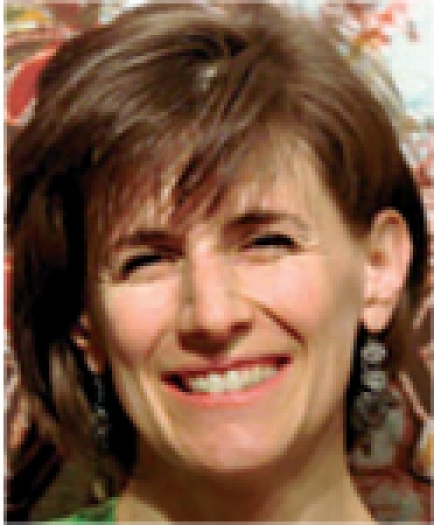


**Figure:**
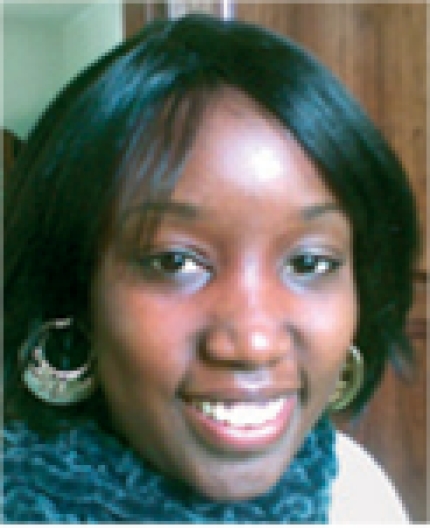


In many low- and middle-income countries, cataract is the leading cause of avoidable blindness among children.^1^

Urgent surgical intervention is necessary if children with cataract are to regain their sight. If children are born with cataracts or if cataracts occur while children are very young, the visual pathways in their brain will not develop normally. Some children may therefore be visually impaired or even blind after their cataracts are removed, especially if there has been a long delay. Fortunately, even if their visual acuity is poor after surgery, most children will regain functional vision; this will enable them to be active and independent.

In order to help children make the best use of the vision they have after cataract surgery, follow-up services are essential. Children may need spectacle correction for near and distance vision as well as low vision devices (optical and non-optical).

Ideally, follow-up should continue for a long time, as children's needs for low vision devices will change as they grow older and want to do more visually challenging tasks. It is also important that potential complications such as thickening of the posterior capsule, development of opacity in the visual axis, glaucoma, or retinal detachment are diagnosed and managed in time.

## Background

In Tanzania, many children are not brought for surgery in a timely fashion and follow up is often poor. Research at Kilimanjaro Christian Medical Centre (KCMC) has shown that girls are more likely than boys to be negatively affected^2^–^4^:

- Only half as many girls as boys received cataract surgery.
- Girls tended to be brought for surgery later than boys.
- Girls who did receive surgery were less likely than boys to be brought for the appropriate two-week follow-up visit (36 per cent of girls vs 64 per cent of boys).

In order to understand why girls were at such a disadvantage, we looked at gender differences in data we had collected during two qualitative studies in Tanzania. In the first study, we had interviewed 117 parents and guardians of children brought for cataract surgery at KCMC between September 2002 and November 2004; our aim had been to uncover why parents sometimes took a long time to bring children for surgery. In the second study, we had conducted interviews with 22 of these parents or guardians, selected for either making good or poor use of follow-up services, in order to understand why follow-up was often poor.

**Figure:**
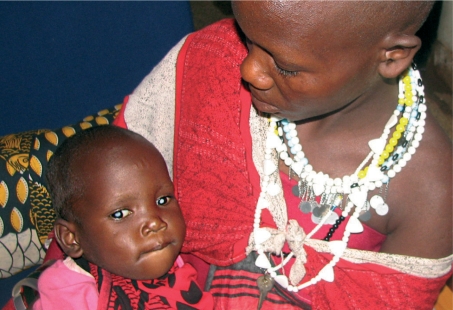
Rosa Isaya Moller (three years old) before her cataract operation. TANZANIA

Elizabeth Kishiki

The reasons parents or carers took a long time to bring children for surgery included the following:

- They did not recognise the disease. Most parents or carers were not aware that a child could have cataract (the same was true for health workers in the communities as well).
- They could not agree on what to do and/or when to do it.
- They had fears about cataract surgery based on mistaken beliefs about what it entailed (for example, that the eye would be removed and then replaced); they also had concerns about the risks associated with surgery and with the stay at the hospital.
- They were concerned about costs (direct and indirect) and the distance they would need to travel.

In general, the reason parents and carers did not bring children for follow-up was because they did not understand that children, unlike most adults, often need low vision devices or spectacles after cataract surgery. They usually saw some improvement of vision after the intervention, and when children could see enough to function, parents were unlikely to consider it necessary to go back for follow-up (which seemed to be true for girls in particular).

## Gender differences

When we analysed the results of both studies according to the gender of the parent and the child, we found the following:

- Fathers (and some mothers) tended to give preference to boys, especially when family resources were scarce.
- Mothers often did not have the power to make decisions about health care for their children; however, those with higher education levels and more financial independence were more likely to be able to influence decisions.
- When asked what they would do if they were able to make such decisions, most mothers wanted to treat their children equally or give preference to daughters over sons.

## A preference for boys

In poor or struggling communities, sons are often seen as a source of income and financial security for parents when they get older, whereas girls are seen as a financial burden. This can mean that boys will be more likely than girls to be taken to a clinic for health care.^5^ It is certainly true in Tanzania, where many families struggle to provide food, shelter, and education for all their children. When difficult choices have to be made, boys often receive preference over girls. The cost of surgery is not the only consideration: time away from work, the need to find someone to look after other children, and long travelling distances need to be considered as well.

From our gender analysis, it was clear that fathers tended to give priority to boys. Fathers often considered that the boy would be able to contribute to family resources and would, in the future, look after his parents.

**Figure:**
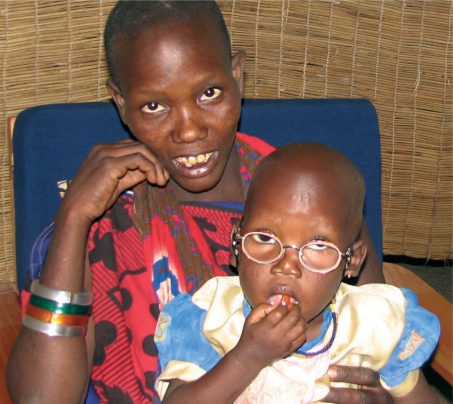
Rose Isaya Moller with her new spectacles, after both cataracts were removed. TANZANIA

Elizabeth Kishiki

“[…] the boy will be responsible for his family while the girl may stay at home with her mother. […] I would send the boy (for surgery) because he will be helpful to me in the future but the girl will be married.” (Interview 17 with Jo's father, primary school, employed in a coffee plantation, waited five years before bringing his son for cataract surgery)

However, choosing boys over girls was not exclusive to fathers. Some mothers did not hesitate to expose their preference for ‘investing’ in a son's health rather than spending money on a girl who would eventually get married and leave.

“I would send the boy.” Q: Why?

A: “Because a boy is more helpful in the society than a girl. ” (Interview 16 with Liz's step mother, peasant, primary school, waited one year before bringing her step daughter for cataract surgery)

## The power to make decisions

Many women are still subject to their husbands' will and wait for his permission to access health care and services for themselves and/or for their children.^5^,^6^

From the 117 interviews we conducted for the first study, it was clear that mothers' influence over the decision making process was closely linked to whether boys or girls were brought for cataract surgery. Less educated women and women with very limited personal financial resources had less capacity to influence decision making.

“I depend on my husband for everything because I am not employed, so I think it is hard to get the money. […] I had to wait for her father to make a decision […] I would have brought her earlier but it is because her father was not ready. ” (Interview 13, with Ma's mother, unemployed, primary school incomplete, waited more than ten years before bringing her daughter for cataract surgery)

Our analysis showed that women's level of education, their socioeconomic status, and the decision-making power they had within their household and their community all played a major role in determining whether and when their children would receive cataract surgery and whether they would be taken for follow-up visits.

We found that, the more educated the parents were (especially mothers), the higher the chances were that:

- a child would be brought for surgery in a timely fashion
- a girl would be brought for surgery (and follow-up), regardless of opposing views from her father
- a child would be brought for post-operative follow-up.^2^–^4^

**What can we do?**

Experience in Tanzania^1^ has suggested that the following strategies can improve access for all children; this will mean that the number of girl children likely to receive cataract surgery will also increase:

- Mass media efforts (especially radio) may provide the first opportunity for rural villagers to learn about the need for early referral of young children with vision loss.
- Many health workers are not familiar with the need for early referral of children with a ‘white pupil’. Brochures and posters have been useful as a continuing medical education tool.
- Cost (direct and indirect) is often the most important barrier preventing use of surgical services by children. Transport reimbursement (for parents and children) is often essential, particularly for parents living quite far away from tertiary hospitals.
- Cell phone penetration has grown significantly in many low- and middle-income countries and phone follow-up with parents has proven to be a very useful strategy for reminding parents to bring children for follow-up visits.

In addition, there are two strategies that will have a more direct impact on improving access for girls:

- Evidence suggests that it is helpful to use key informants to identify and refer children who need eye care. More girls are identified this way than when relying on parents alone to recognise the need for surgery.
- Paediatric ophthalmology tertiary facilities can benefit from having a dedicated childhood blindness coordinator who can provide high quality counselling and support services for parents and guardians. With the help of such a coordinator, parents learn the benefits of early surgery, follow-up, and rehabilitation and become more engaged in the care of their children, particularly girls. Using a tracking form (showing when the operation was performed and all follow-up dates) helps the coordinator to monitor follow-up, counselling needs, and patient information efforts. In order to support the coordinator's work, it is important to link clinical services at paediatric ophthalmology units with general ophthalmology units and other eye care providers as well as with educational and rehabilitation services.

1KishikiKShirimaSLewallenSCourtrightPImproving postoperative follow-up of children receiving surgery for congenital or developmental cataracts in AfricaJ of AAPOS(in press).10.1016/j.jaapos.2008.12.00219285887
